# Editorial: Leading People – Managing Organizations: Contemporary Public Health Leadership

**DOI:** 10.3389/fpubh.2015.00268

**Published:** 2015-11-25

**Authors:** James W. Holsinger, Erik L. Carlton, Emmanuel D. Jadhav

**Affiliations:** ^1^University of Kentucky, Lexington, KY, USA; ^2^University of Memphis, Memphis, TN, USA; ^3^Ferris State University, Big Rapids, MI, USA

**Keywords:** editorial, public health leadership, public health management

Effectively leading people engaged in the practice of public health has never been more critical than in the early years of the twenty-first century. Likewise, effectively managing the organizations in which these individuals practice the various professional disciplines of public health has become increasing important and difficult. Taken together, leading the people and managing public health organizations requires well educated and appropriately trained public health leaders and managers. Although leadership is often viewed as one of the key attributes of management, not every great manager will be a great leader and vice versa. While some leaders may be born with the inherent skills to lead, most effective leaders develop the requisite skills through education, additional training, and practice.

Our aim is to focus the attention of public health practitioners on the importance of effectively leading public health organizations. Public health managers should recognize that their most valuable resource is the people they lead. The articles comprising the eBook on Leading People – Managing Organizations is composed of articles expressing the opinion of their authors of the need for effective public health leaders; perspective articles establishing their authors’ understanding of how leadership may be applied in various situations; methods articles that demonstrate how public health leadership may be applied, and original research articles that establish the role of public health leadership research studies.

## Opinion

Baroff, Yphantides et al., and Van Gorder express their opinions on the need for effective public health leadership based on their personal experiences in public health practice. Baroff (1) details the development of her career in leading organizations, as well as the difficulties she encountered in doing so. Her opinion clearly indicates the need for persistence and self-awareness for all leaders. Yphantides et al. (2) issue a call for public health leaders to develop new leadership skills in order to implement and sustain change within the public health organizations they lead. Their article summarizes their understanding of the need for new competencies that are essential for moving the public health system forward in the twenty-first century. Van Gorder (3) calls for public health and healthcare leaders to work together in order to build strong collaborative models for the future.

## Perspective

Rabarison et al. (4) provide their perspective on utilizing Situational Leadership^®^ as an effective leadership process in developing public health agency accreditation in the USA through the national voluntary public health accreditation process. Utilization of this contingency theory of leadership allows public health practitioners and staff members to develop the knowledge and confidence required to meet the standards of the Public Health Accreditation Board. Carman (5) provides a perspective on the impact that public health agency and academic institutional partnerships have on the public health accreditation process in the USA. She identifies the opportunity for academic institutions to provide consultative services to public health agencies in an effort to arrive at a successful conclusion to the accreditation process. Carman (6) further provides an interesting perspective on the application of leadership and followership theories to voluntary public health agency accreditation in the USA. She proposes that “teamship” rather than leadership or followership is required in order to create an accreditation readiness team prepared to guide a local health department through the accreditation process. Rabarison et al. (7) provide an interesting perspective on the need for evidence-based public health practices and the resulting decision making by public health leaders as they consider the impact of reduced funding and constrained budgets. They contend that population health is optimized as public health leaders identify, measure, and compare the activities being conducted by public health agencies with their resulting impact, scalability, and sustainability. Public health leaders need to conduct economic evaluation of public health activities in order to make appropriate decisions affecting the health of the population being served. From an international perspective, Negandhi et al. (8) identified interdisciplinary leadership competencies among health practitioners, such as self-awareness, vision, self-regulation, motivation, decisiveness, integrity, interpersonal communication skills, strategic planning, team building, innovation, and functioning as an effective change agent. Their pilot study in India developed a training model for building such skills through interdisciplinary workshops with the objective of incorporating such training in the medical, nursing, and public health curricula. They propose through the use of transformative learning that leadership skills be incorporated into healthcare professional training in a variety of national contexts.

## Methods

Marckmann et al. (9) provide a systematic framework for putting public health ethics into practice. Public health practice requires a different approach to ethical concerns than that of traditional biomedical ethics. They propose two necessary components to practicing public health ethics: a set of normative criteria that are based on an explicit ethical justification and a structured methodological approach for applying these criteria to specific public health issues. They recommend that their framework be put in practice in public health settings in an effort to determine its practical application.

## Original Research

A group of four original research articles rounds out the research topic. In an investigation into the impact of emotional intelligence on the conditions of trust found in a public health setting, Knight et al. (10) measured emotional intelligence including stress management among supervisors in the Kentucky Department of Public Health (USA). The study found significant positive correlations between supervisors’ stress management and the staff members’ trust or perception of the supervisors’ loyalty, integrity, receptivity, promise fulfillment, and availability. Findings such as these provide the requisite tools to provide training opportunities related to emotional intelligence and trust in organizations. In a two-part article, Carlton et al. (11, 12) consider full-range public health leadership as a useful construct for considering the complex challenges faced by effective public health leaders. They provide both a quantitative as well as a qualitative analysis and synthesis utilizing transformational leadership as a model for their study. They determined that transformational and transactional styles of leadership need to be balanced in order to provide effective leadership to public health organizations. As a result, both approaches have beneficial results depending on the context or situation in which they are utilized. When leaders lead by example and are collaborative, transformational leadership is effective. However, there are occasions when a transactional style of leadership is required to assure adequate performance levels and the accomplishment of certain tasks. Jadhav et al. (13) studied openness to change on the part of local health department leaders (USA). They demonstrated that leaders had relatively high openness to change scores based on their understanding of the characteristics of an innovative strategy. Their analyses found important relationships between the characteristics of the leader and those of the public health agency on the leader’s openness to change.

## Summary

The articles composing the *Leading People – Managing Organizations* research topic approach effective public health leadership from a variety of viewpoints. Together these opinion, perspective, method, and original research articles point to the need for further development of public health leadership in the twenty-first century. Utilizing diverse leadership theories or models, as well as considering the needs expressed in the opinion and perspectives articles by authors engaged in public health practice and applied public health services research, additional research is needed to develop evidence-based approaches to the practice of effective public health leadership in the twenty-first century.

## Author Contributions

JH wrote the original draft of the article. EC and EJ revised and corrected the original draft.

## Conflict of Interest Statement

The authors declare that the research was conducted in the absence of any commercial or financial relationships that could be construed as a potential conflict of interest.
